# Global prevalence of post-abortion depression: systematic review and Meta-analysis

**DOI:** 10.1186/s12888-023-05278-7

**Published:** 2023-10-26

**Authors:** Natnael Atnafu Gebeyehu, Kirubel Dagnaw Tegegne, Kelemu Abebe, Yibeltal Asefa, Belete Birhan Assfaw, Getachew Asmare Adella, Biresaw Wassihun Alemu, Dagne Addisu Sewyew

**Affiliations:** 1 School of Midwifery, College of Medicine and Health Sciences, Wolaita Sodo University, Sodo, Ethiopia; 2 Department of Comprehensive Nursing, College of Medicine and Health Science, Wollo University, Dessie, Ethiopia; 3 Department of Public Health, College of Medicine and Health Science, Wolaita Sodo University, Sodo, Ethiopia; 4 Department of Psychiatry, College of Medicine and Health Science, Wolaita Sodo University, Sodo, Ethiopia; 5 Department of Reproductive Health, College of Medicine and Health Science, Wolaita Sodo University, Sodo, Ethiopia; 6 Department of Midwifery, College of Medicine and Health Science, Indiana University, Injibara, Ethiopia; 7 Department of Midwifery, College of Medicine and Health Science, Debre Tabor University, Debre Tabor, Ethiopia

**Keywords:** Post-abortion, Depression, Global, Systematic review, Meta-analysis

## Abstract

**Background:**

Depression after abortion is a common problem for all women of reproductive age. However, there are not any data on post-abortion depression at a global level. Consequently, the purpose of this study was to find out the global prevalence of post-abortion depression.

**Methods:**

The present study involved a comprehensive search of several databases, including Science Direct, Scopus, EMBSE, Google Scholar, and PubMed. The search was conducted between February 1, 2023, and March 10, 2023. The data was extracted using Microsoft Excel (version 14) and analyzed using STATA statistical software. To evaluate publication bias, a forest plot, Begg’s test, and Egger’s test were employed. Heterogeneity was assessed using I^2^, and a pooled estimated analysis was conducted. Additionally, subgroup analysis was performed based on the study continent/region, World Bank income group, screening instrument, and study design.

**Results:**

This analysis included 15 papers with a total of 18,207 research participants out of a total of 657 articles. The overall pooled prevalence of post-abortion depression was found to be 34.5% (95% CI: 23.34, 45.68), with an I^2^ value of 71.6%. The prevalence of post-abortion depression varied based on geographic location, World Health Organization (WHO) regions, World Bank income category, screening approach, and study design. The highest proportion of post-abortion depression was observed in Asia (37.5%), while the WHO’s Eastern Mediterranean region had the greatest rate of post-abortion depression (43.1%). Lower-middle-income countries had the highest frequency of post-abortion depression (42.91%) based on World Bank economic classification. The Center of Epidemiological Studies Depression Scale was found to have the highest incidence of reported depression prevalence (30%) across diagnostic tools. Furthermore, the prevalence of depression was higher in cross-sectional study designs (36.42%) compared to cohort studies (22.7%).

**Conclusion:**

In conclusion, the occurrence of post-abortion depression has been observed to be widespread globally. The prevalence of post-abortion is found to be influenced by several factors, including the methodology employed in the study, the diagnostic tool utilized, the geographical location, and the socioeconomic status of the population. Healthcare providers should prioritize the provision of post-abortion counseling, care, and emotional support to women.

**Supplementary Information:**

The online version contains supplementary material available at 10.1186/s12888-023-05278-7.

## Introduction

Pregnancy and childbirth-related complications are a primary cause of mortality and morbidity among women of reproductive age [1]. Abortion, a complication that can occur during both early and late pregnancy, is a significant issue affecting approximately one in four women [2]. Clinically diagnosed pregnancies result in abortion in 10–15% of cases, while 60% of all pregnancies end in abortion [3]. The experience of pregnancy loss can have a profound impact on women’s physical, psychological, and mental well-being [4]. Women who experience pregnancy loss often report feelings of distress and anxiety, which can have a significant emotional impact on both themselves and their partners [5]. Depression is a major public health concern, with women being twice as likely as men to experience depression during their lifetime [6]. Depression is a leading cause of disability worldwide [7], and approximately one in five women experience depression after giving birth [8]. Furthermore, the incidence of severe post-abortion depression is three times higher than during other periods of women’s lives [9].

During the occurrence of a miscarriage, a majority of women are likely to undergo a period of intense emotional distress [10], which can manifest in various symptoms of grief, including but not limited to sadness, yearning, social isolation, and guilt [11]. It is important to note that the impact of such an event on a woman’s life may be erroneously underestimated [12]. Many women may hold themselves personally responsible for the miscarriage [13], which can exacerbate feelings of self-blame and lead to heightened levels of anxiety, depression, and post-traumatic stress disorder [13]. Unfortunately, some women may not receive adequate screening for depression, which can leave them unidentified and untreated, thereby increasing the risk of psychiatric squealed [14].

The occurrence of psychiatric morbidity in women following a miscarriage has a discernible impact on whether the miscarriage is spontaneous or induced [15]. Research has indicated that induced miscarriages are associated with higher rates of psychological issues compared to spontaneous ones [16]. A variety of mental morbidities, including depression, have been linked to psychiatric disorders, which have been identified as a significant cause of miscarriages [17, 18]. Women who have experienced a miscarriage may encounter depression at different stages [19]. The depressive disorder appears to be a significant burden following a miscarriage, with symptoms emerging as early as 10 days and potentially persisting for a lifetime [20]. Furthermore, women who undergo abortions come from diverse socio-cultural backgrounds, in contrast to those who carry a fetus to term [21].

Works of literature showed that the incidence of depression following a miscarriage is significantly higher in women who have experienced such a loss compared to those who have not [21]. Depression-related symptoms, such as exhaustion, lack of enjoyment, and low self-esteem, can impede sexual function, while sexual dysfunction can indirectly lead to infertility by reducing the frequency of sexual encounters [22, 23]. Fabre and Smith’s study indicates that women’s sexual dysfunction worsens as their depression becomes more severe [24]. The findings of a study conducted in Australia suggest that the presence of any risk factors, such as anxiety, depression, and sexual dysfunction, increases the likelihood of one or more future disorders [25]. Depression is closely associated with decreased libido, dyspareunia, and orgasmic dysfunction [26, 27]. Even in the absence of clinical signs of depression, a negative mood can cause sexual dysfunction [28, 29].

The data indicates a notable shift in the prevalence of post-abortion depression over the past decade. Specifically, the prevalence of this condition decreased from 30% [30] in 2008 to 8.6% [31] in 2018. However, recent trends suggest a concerning increase in depression rates, with a rise from 37% [32] in 2019 to 48.6% [33] in 2021.

Numerous primary studies have been conducted globally to determine the prevalence of post-abortion depression [30–44]. These independent studies have revealed a significant variation and inconsistency in the prevalence of post-aborted depression worldwide, with estimates ranging from 8.6% [31] to 85% [44]. The heterogeneity in post-abortion depression prevalence among women of reproductive age noted above necessitates pooling and utilizing this information on an international level. Furthermore, the current understanding of the epidemiology of depression is primarily based on a limited number of regional surveys and insufficient national data. To address this gap, the present study aims to update the epidemiology of post-abortion depression and provide evidence-based information to prioritize mental health therapy for mothers.

## Methods

### Reporting

The present study adhered rigorously to the checklist outlined by the Preferred Reporting Items for Systematic Reviews and Meta-Analysis (PRISMA) guidelines [45] (Additional file 1). The protocol for this systematic review and meta-analysis has been submitted to the International Prospective Register of Systematic Reviews (PROSPERO) under registration number CRD42023415343.

### Search strategy

The utilization of modified PICO questions was considered, wherein the “PEO” (Population, Exposure, Outcome) format was employed to explicitly present our review inquiry and to clarify the criteria for inclusion and exclusion. These inquiries were constructed through the amalgamation of specific keywords and phrases and/or Medical Subject Headings (MeSH), in conjunction with the Boolean operators “OR” and “AND”.

### PECO guide

#### Population

All reproductive-age women with an abortion history.

#### Exposure

Post-abortion mothers who considered depression as screened by depression screening tools.

#### Outcome

##### Depression

We developed the following review question using the above modified PICO format, intending to identify as many relevant primary studies as possible:

###### Review question

“What is the global prevalence of post-abortion depression among women?”

The current study utilized international web databases, namely Pub Med, Science Direct, Scopus, EMBASE, and Google Scholar, to conduct a comprehensive search for articles about the prevalence of post-abortion depression on a global scale. The search was conducted between February 1, 2023, and March 10, 2023, and employed a range of search terms and keywords, including “prevalence”, “magnitude”, “proportion”, “depression”, “depressive symptoms”, “emotional depression”, “depressive disorder”, “psychological distress”, “abortion”, “post-abortion”, “miscarriage”, “induced abortion”, “safe abortion”, “unsafe abortion”, “legal abortion,“ “illegal abortion,“ and “criminal abortion”. These search terms were used both independently and in combination, utilizing Boolean operators such as “OR” and “AND”.

### Inclusion and exclusion criteria

Those articles were included in this systematic review and meta-analysis of (1) Study type: All observational studies reporting the prevalence of post-abortion depression (2) Population: Studies done among post-aborted women (3) Language: English (4) Place of study: Globally (5) The full text was available during searching. This systematic review and meta-analysis omitted all qualitative studies, letters to the editor, comments, expert opinions, case studies, case series, and randomized control trials.

### Quality assessment

This study employed a standardized quality appraisal checklist developed by the Joanna Briggs Institute (JBI) [46] (Additional file 2) to evaluate the level of research. Two authors NAG and KDT independently conducted the appraisal. The critical analysis checklist comprised eight parameters, each with yes, no, uncertain, and not relevant boxes. The parameters included inquiries such as the clarity of inclusion criteria for the sample, the thoroughness of the description of study participants and the environment, the validity and reliability of exposure measurement results, the meeting of primary purpose and accepted standards, the identification of confounding elements, the mention of confounding factor measures, the accuracy of outcome measurement, and the appropriateness of statistical analysis. Disagreements that arose during the quality assessment were resolved through a dialogue facilitated by the third author, DAS. Studies that scored 50% and above on the quality assessment indicators were considered low risk. The agreement between the two reviewers was assessed using their actual agreement and agreement that was not just a coincidence (Kappa). A Kappa value of 0 is regarded as having poor agreement, 0.01 to 0.02 as having only a small agreement, 0.21 to 0.4 as having a fair agreement, 0.41 to 0.60 as having a moderate agreement, 0.61 to 0.80 as having a large agreement, and 0.81 to 1.00 as having practically perfect agreement. In this review, a nearly perfect agreement was found, with the real agreement beyond chance falling between 0.88 and 1.

### Risk of bias assessment

This research employed the bias assessment tool developed by Hoy et al. [47], which comprises ten items that evaluate four domains of bias, as well as internal and external validity. Two authors, NAG and DAS, independently assessed the included papers for potential bias. Any discrepancies that arose during the risk of bias assessment were resolved through a discussion led by the third author, KDT. Ultimately, a consensus was reached through this process. The first four items of the tool (items 1–4) pertain to the assessment of selection bias, non-response bias, and external validity. The remaining six items (items 5–10) evaluate the presence of measurement bias, analysis-related bias, and internal validity. Studies were categorized as having a “low risk of bias” if they answered “yes” to eight or more of the ten questions. Studies were classified as having a “moderate risk” if they answered “yes” to six to seven of the ten questions, while studies that answered “yes” to five or fewer of the ten questions were classified as having a “high risk” of bias (Additional file 3).

### Data extraction

Data extraction and analysis were carried out using STATA 14 software and a Microsoft Excel spreadsheet from 2016 respectively. To ensure consistency and accuracy, a standardized Joanna Briggs Institute data extraction format was utilized by two authors (NAG and KDT) who independently extracted all relevant data. Any discrepancies that arose during the data extraction process were resolved through a discussion led by the third author (DAS). Ultimately, a consensus was reached among the authors. The extracted data included the first author’s name, year of publication, study region, study setting, study design, sample size, prevalence of post-aborted depression, depression screening tool, and quality of each paper.

### Data analysis

The data extracted from a Microsoft Excel spreadsheet (2016) were exported to STATA software version 14 for analysis. In the field of data pooling, two common approaches are typically employed: the two-step method and the one-step method. The former involves a process of data cleaning, followed by the application of a standard or widely accepted cut-off value for each scale. In the context of assessing depressive status, this method involves dichotomizing each participant’s status as either ‘yes’ or ‘no’, and subsequently computing the prevalence of depression for the study. To combine prevalence data from multiple studies, a two-step method is often utilized. This involves extracting the total number of participants and events from each study and subsequently utilizing a random-effects model to combine the prevalence data through the use of STATA statistical software. There are various techniques available to alleviate the effects of publication bias, such as (1) registering study protocols before initiating research, (2) employing funnel plots to visually assess the likelihood of publication bias, and (3) executing a comprehensive search strategy to investigate diverse grey literature sources.

Subgroup analyses were conducted based on the continent of the study, study design, and measurement methods employed. Furthermore, sub-group analyses were performed for each of the WHO regions, including Africa, America, South-East Asia, Europe, Eastern Mediterranean, and Western Pacific, as well as the World Bank income categories, namely low, lower middle, higher middle, and high income. Sensitivity analysis was employed to determine the impact of a single study on the overall meta-analysis estimate of prevalence. The funnel plot was used to examine potential publication bias, and Begg and Egger’s regression tests were employed to examine it more objectively. Cochran’s Q X^2^ test and I^2^ statistics were utilized to test for heterogeneity, estimate the amount of total/residual heterogeneity, and measure variability caused by heterogeneity, respectively [48]. A Univariate meta-regression analysis was conducted to examine the effects of sample size and publication year variations on between-study heterogeneity [49].

## Results

### Search results and study characteristics

**Fig. 1:**
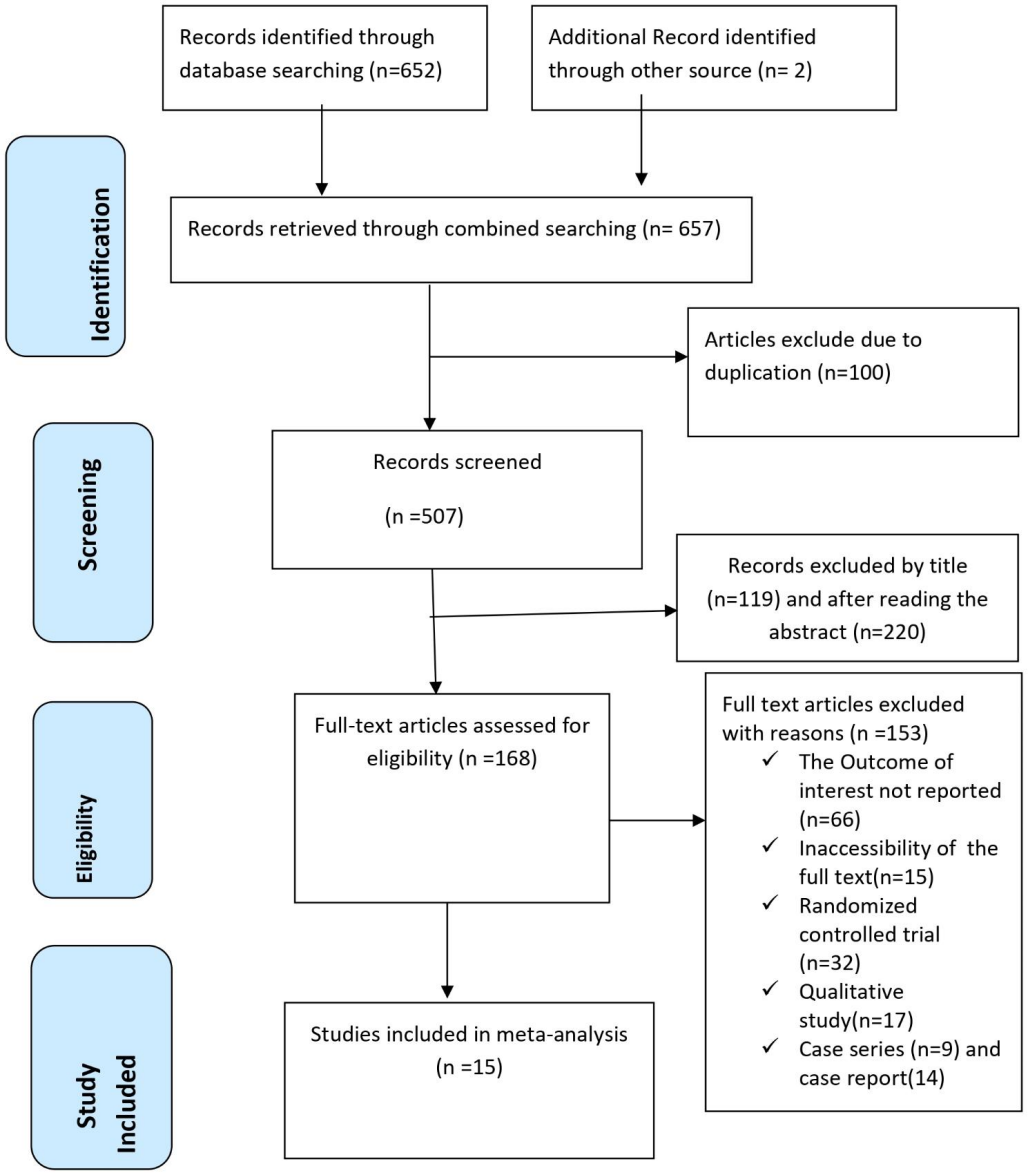
PRISMA flow chart displays the article selection process for the global prevalence of post-abortion depression

Initially, a total of 657 studies were identified through our search approach across various electronic resources worldwide. Following the removal of 100 duplicate papers, 507 studies remained. Upon reviewing the titles and abstracts, we identified 168 papers that were relevant to the research question. Subsequently, after a thorough examination of the full articles, we excluded 153 of these papers for various reasons. Ultimately, 15 studies [30–44] comprising 18,207 study participants were deemed eligible for inclusion in this systematic review and meta-analysis study (Fig. 1).

The present study analyzed a total of 15 studies, of which the majority (11; [33, 35–39, 43, 44]) were cross-sectional, followed by three cohort studies [30, 34, 41] and one case-control study [42]. Specifically, 14 studies (93%) were cross-sectional, while only one (0.7%) was community-based. The geographic distribution of the studies was as follows: three studies were conducted in China [39, 41, 42], two in Germany [37, 41], two in Iran [38, 43], and one study each in Australia [30], Kenya [31], Netherlands [34], Jordan [35], Kosovo [36], Denmark [39], Lithuania [40], and Turkey [44]. The measurement of depression was assessed using various instruments across the studies. Specifically, five studies employed the Edinburgh Postnatal Depression Scale, while three studies utilized the PHQ-9 tool for screening depression. Two studies used the Self-Rating Depression Scale, one study employed Beck’s inventory scale, and another study used the Hospital Anxiety Depression Scale. Two studies utilized the Center of Epidemiological Studies Depression Scale, while one study did not specify the type of instrument used to measure depression. Sample sizes varied widely, ranging from 60 to 12,158 participants. The prevalence of post-abortion depression ranged from 8.6 to 85%. Overall, all studies included in the analysis were deemed to have low risk (Table 1).

**Table 1:** Characteristics of studies included in the systematic review and meta-analysis of post-aborted depression

Author/year	Country	Setting	Design	Sample size	Prevalence	Measurement scale	Quality
A.A Boersma/2014 [34]	Netherland	Institutional	Cohort	92	30%	CES-D	Low-risk
Akdag Topal/2019 [44]	Turkish	Institutional	Cross-sectional	60	85%	HADS	Low-risk
Angela J Taft/2008 [30]	Australia	Community	Cohort	1076	30%	CES-D	Low-risk
Asma Sa’d Basha et.al/2020 [35]	Jordan	Institutional	Cross-sectional	200	22.5%	PHQ-9	Low-risk
F.Hanschmidt et.al/2017 [37]	Germany	Institutional	Cross-sectional	148	10.8%	PHQ-9	Low-risk
Bujar Obertina et.al/2016 [36]	Kosovo	Institutional	Cross-sectional	122	27.8%	EPDS	Low-risk
Farnoosh Moafi/2018 [38]	Iran	Institutional	Cross-sectional	185	54%	EPDS	Low-risk
Kolte et.al/2014 [39]	Danish	Institutional	Cross-sectional	301	8.6%	SDS	Low-risk
Kukulskiene/ 2016 [40]	Lithuania	Institutional	Cross-sectional	839	59.1%	EPDS	Low-risk
L.Gao et.al/2019 [32]	China	Institutional	Cross-sectional	278	37%	EPDS	Low-risk
L.Jacob et.al/2017 [41]	Germany	Institutional	Cohort	12,158	8.9%	NR	Low-risk
Zhang et.al/2021 [33]	China	Institutional	Cross-sectional	253	22.5%	PHQ-9	Low-risk
Wang et.al/2021 [42]	China	Institutional	Case-control	1132	48.6%	SDS	Low-risk
Azin et.al/2020 [43]	Iran	Institutional	Cross-sectional	130	40.8%	Beck’s scale	Low-risk
Mutiso et.al/2018 [31]	Kenya	Institutional	Cross-sectional	182	8.6%	EPDS	Low-risk

### Meta-analysis

#### Global prevalence of post-abortion depression

**Fig. 2:**
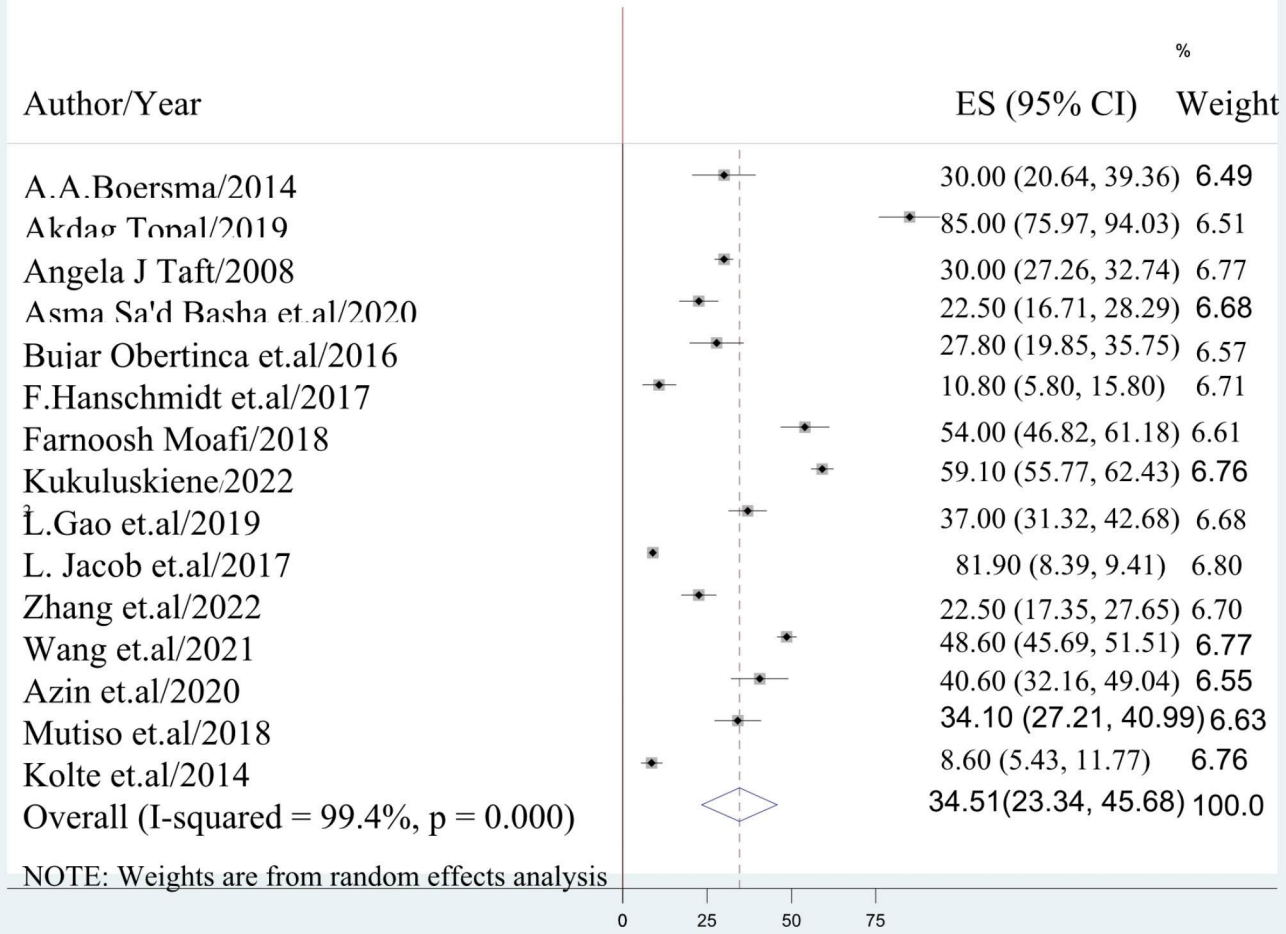
Forest Plot displaying global pooled prevalence of post-abortion depression

The present study employed a random-effects model to derive the aggregate estimate of post-abortion depression. The findings revealed that the worldwide prevalence of post-abortion depression was estimated to be 34.5% (95% CI: 23.34, 45.68), with a corresponding heterogeneity score of (I^2^) = 99.4% **(**Fig. 2).

### Subgroup analysis

Based on region/continent, the six WHO regions, the World Bank region, depression screening, and study design, subgroup analysis was carried out.

#### The prevalence rate of post-abortion depression based on continents

**Fig. 3:**
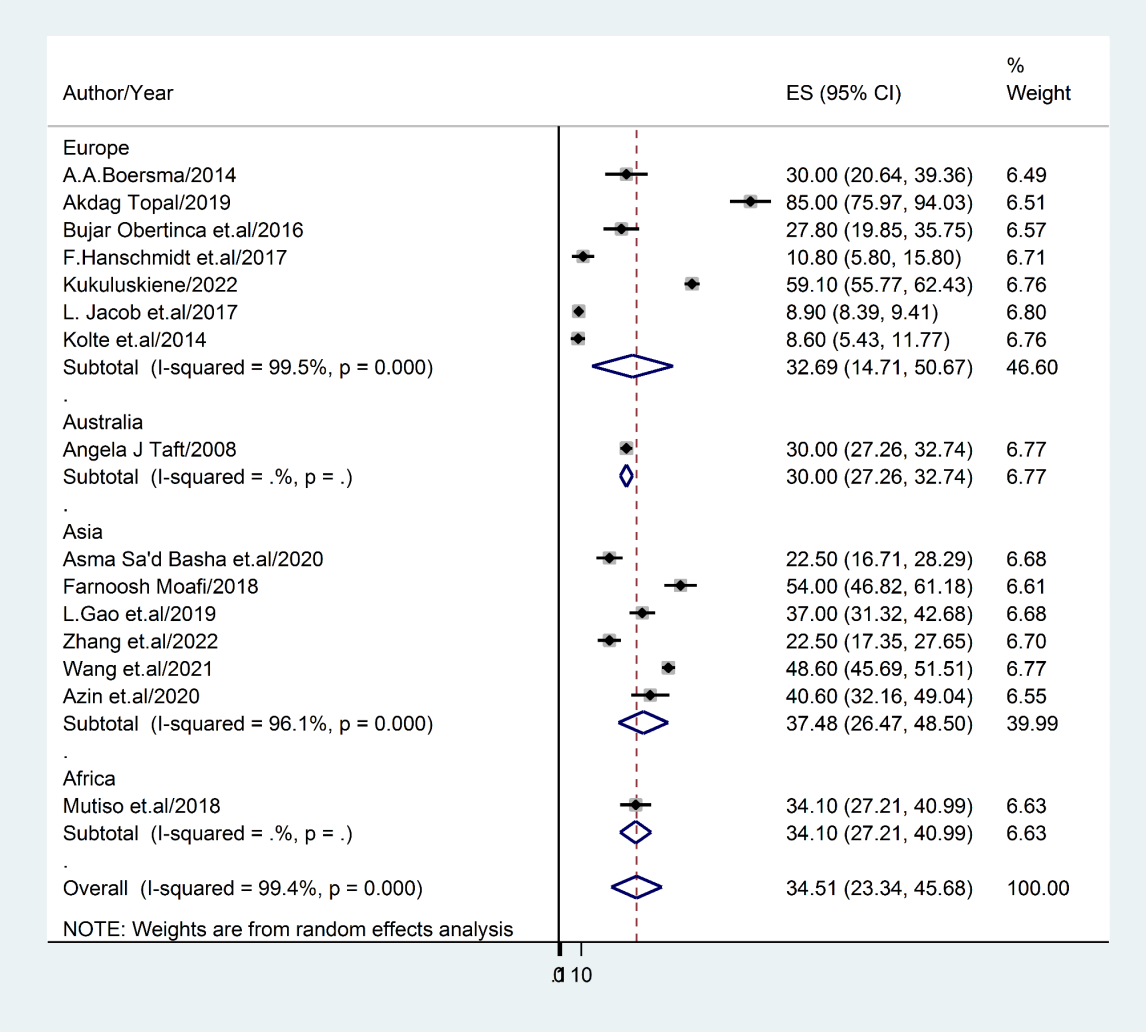
Forest plot displaying sub-group analysis of post-abortion depression based on continent

A sub-group analysis was performed based on continents due to the presence of significant heterogeneity. The results indicated that Asia exhibited the highest prevalence of post-abortion depression (37.48%; 95% CI: 26.47, 48.50; I^2^ = 41.7%), while Europe exhibited the lowest prevalence (32.69%; 95% CI: 14.71, 50.67; I^2^ = 52.3%) (Fig. 3**).**

#### The prevalence rate of post-abortion depression based on six WHO regions

**Fig. 4:**
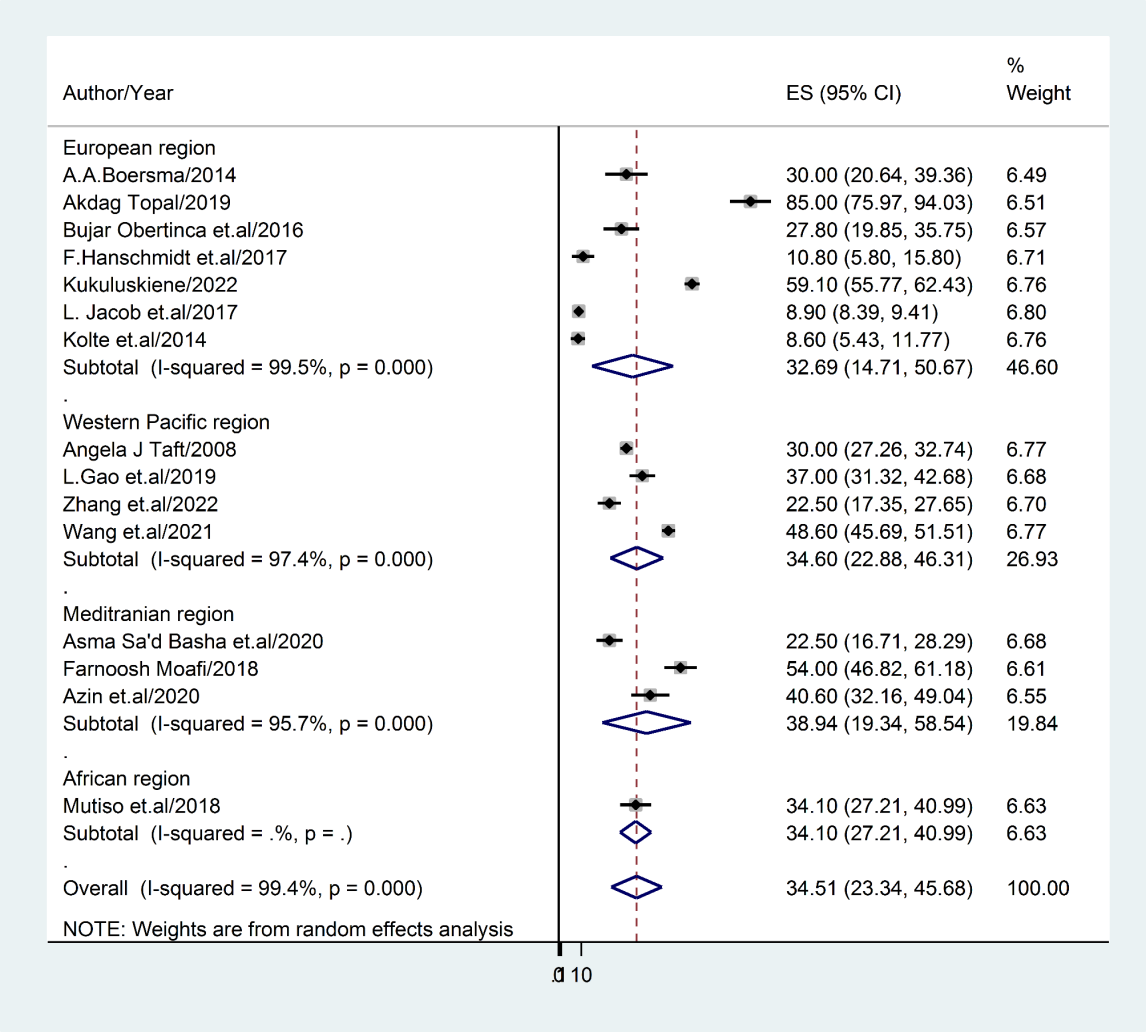
Forest plot displaying sub-group analysis of post-abortion depression based on WHO regions

This study aimed to investigate the prevalence rate of post-abortion depression across six World Health Organization (WHO) regions. The Mediterranean region exhibited the highest prevalence rate at 43.1% (95% CI: 19.34–58.54), I^2^ = 49.3%, while the European region had the lowest prevalence rate at 32.7% (95% CI: 14.71–50.67), I^2^ = 35.7%. Notably, the WHO Regional Office for the South East Asia and Region of the Americas has not conducted a study on this topic to the best of our knowledge (Fig. 4).

#### The prevalence rate of depression based on income status

**Fig. 5:**
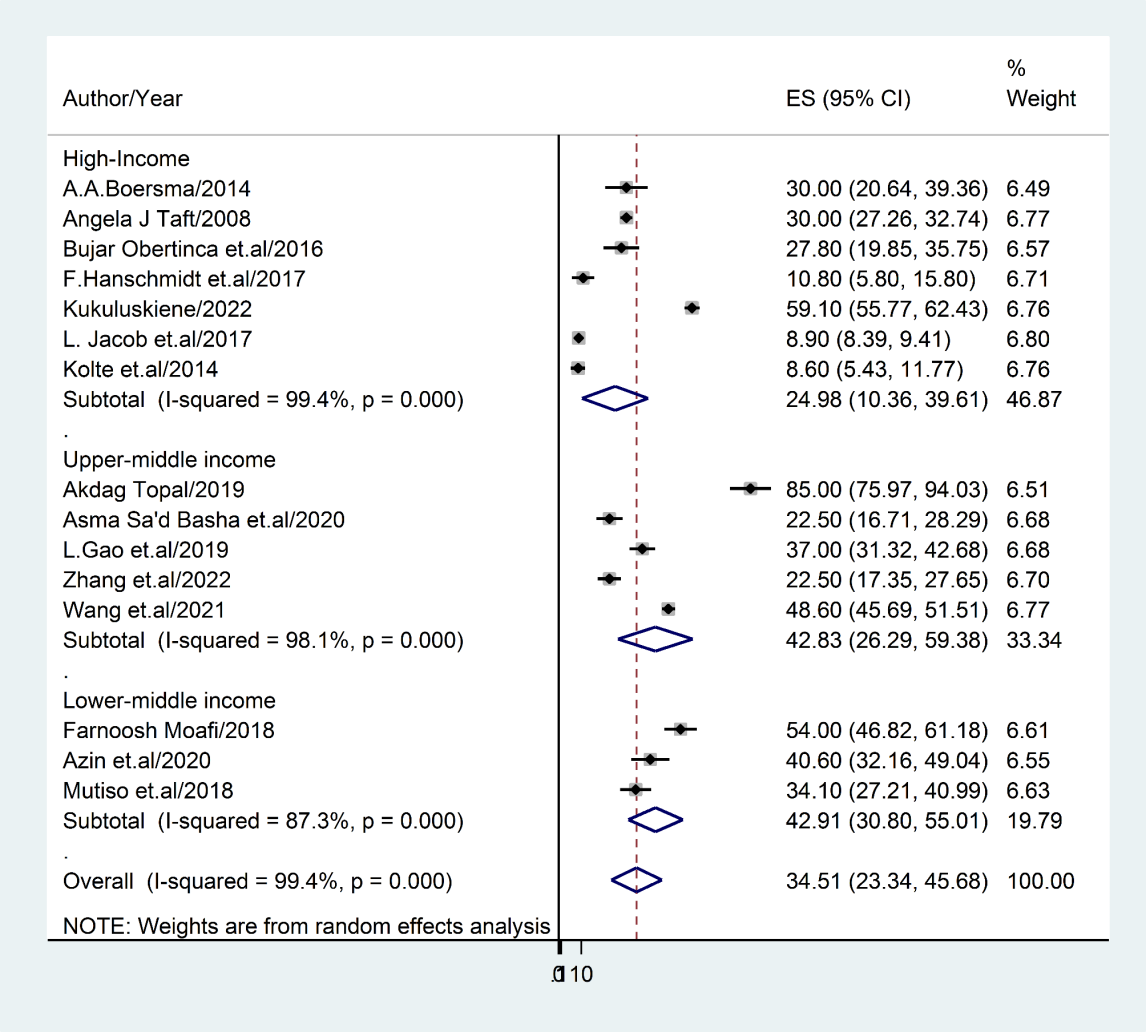
Forest plot displaying sub-group analysis of post-abortion depression based on World Bank income group

Utilizing the World Bank’s assessment of national income levels, a sub-group analysis was performed. Our investigation revealed that lower-middle-income nations exhibited a greater incidence of post-abortion depression (42.91%:95%CI: 30.80-55.01), I^2^ = 38.3%, in comparison to high-income countries (24.98%:95%CI: 10.36: 39.61), I^2^ = 21.3% **(**Fig. 5**).** Notably, no prior research has been conducted in low-income countries as classified by the World Bank.

#### The prevalence rate of depression based on study tools

**Fig. 6:**
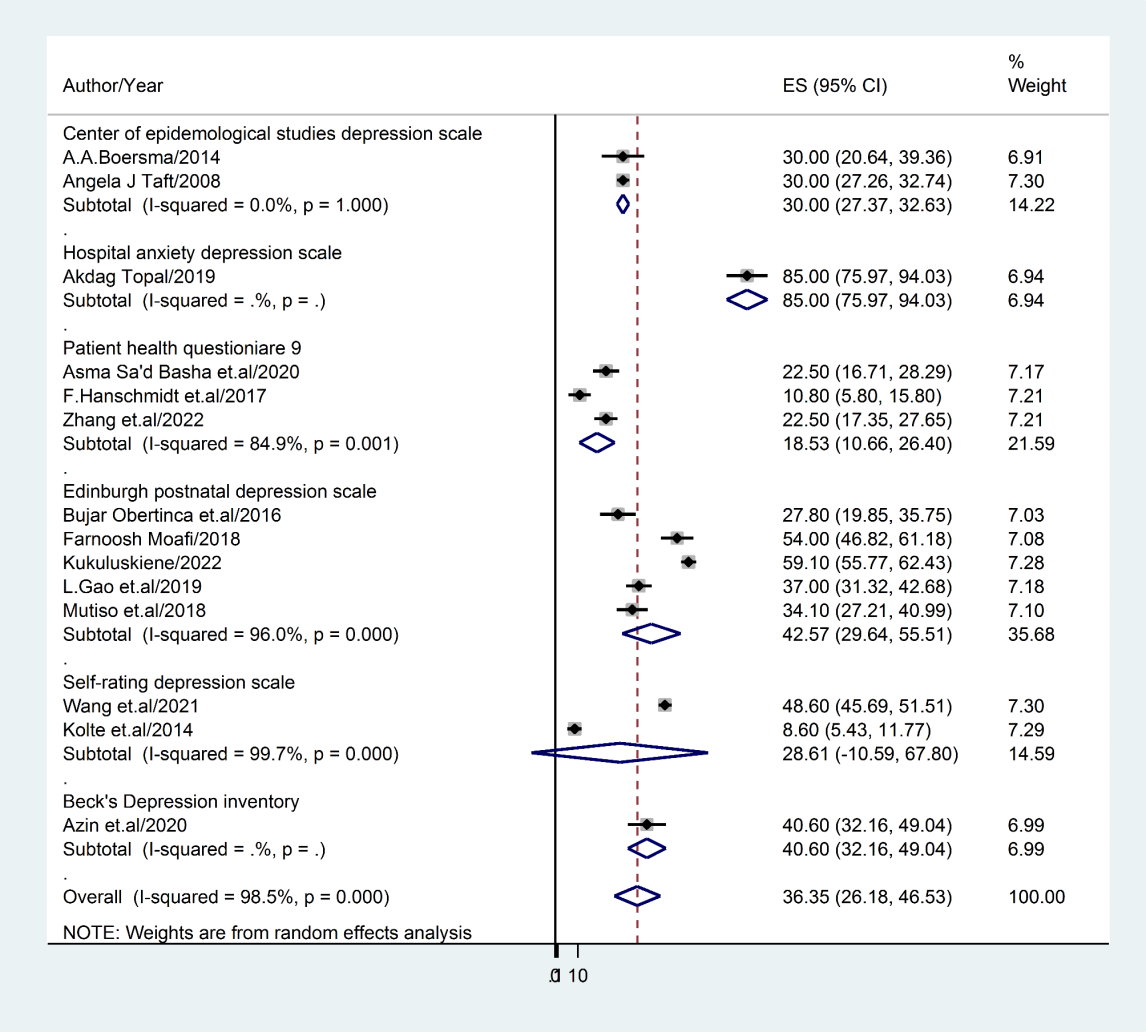
Forest plot displaying sub-group analysis of post-abortion depression based on screening tools

Various measurement scales were employed to assess depression. As a result, the Center of Epidemiological Studies Depression Scale (with a prevalence of 30% and a 95% confidence interval of 27.37–32.63), exhibiting an I^2^ value of 12.8%, and the Edinburgh Postnatal Depression Scale (with a prevalence of 18.53% and a 95% confidence interval of 10.66–26.40), exhibiting an I^2^ value of 17.3%, demonstrated the highest and lowest rates of depression, respectively (Fig. 6).

#### Prevalence of depression based on study design

**Fig. 7:**
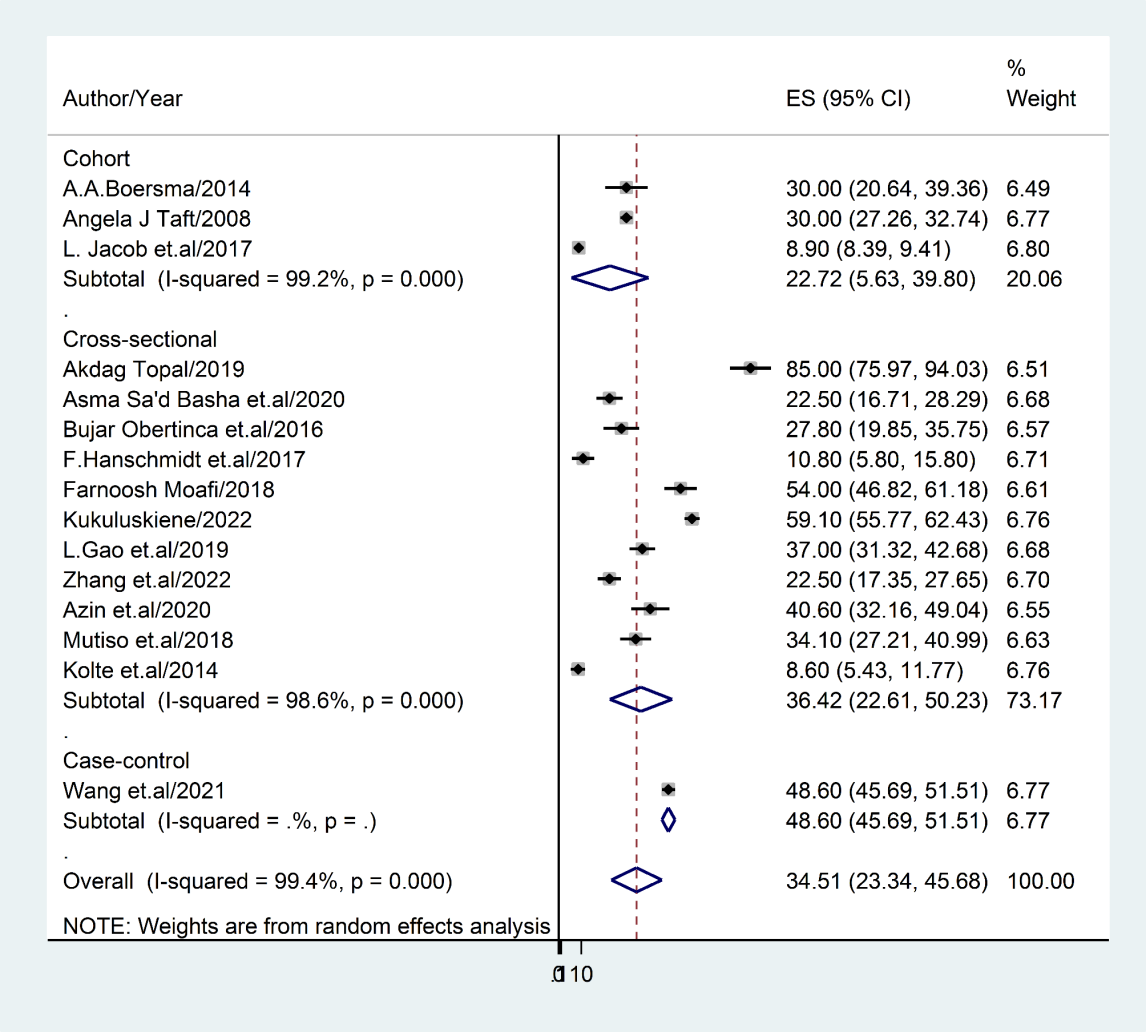
Forest plot displaying sub-group analysis of post-abortion depression based on World study design

This meta-analysis has produced results that suggest a prevalence rate of 36.42% (95% [CI]: 22.61–50.23) for post-abortion depression in cross-sectional studies, with an I^2^ statistic of 31.3%. In cohort studies, the estimated prevalence rate for post-abortion depression was 22.72% (95% CI: 5.63–39.80), with an I^2^ statistic of 27.5 **(**Fig. 7).

#### Heterogeneity and publication bias

In light of the region/continent, study methodology, and measurement utilized to address the purported heterogeneity of the study (I^2^ = 99.4%), our conclusion was drawn through a sub-group analysis. Additionally, a univariate meta-regression analysis was conducted, with sample size, publication year, continent/region, and study design serving as covariates, to identify the primary sources of heterogeneity. The results indicated that continent (p = 0.014) and sample size (p = 0.000) significantly impacted the variability observed across the studies (Table 2).

**Table 2:** Meta-regression analysis of factors affecting between-study heterogeneity

Heterogeneity source	Coefficient’s	Standard error	p-value
Year	91.09147	258.6252	0.35
Sample size	3.406128	0.6552413	0.000
Continent	5.057477	1.795266	0.014
Study design	1.816004	1.789734	0.327

**Fig. 8:**
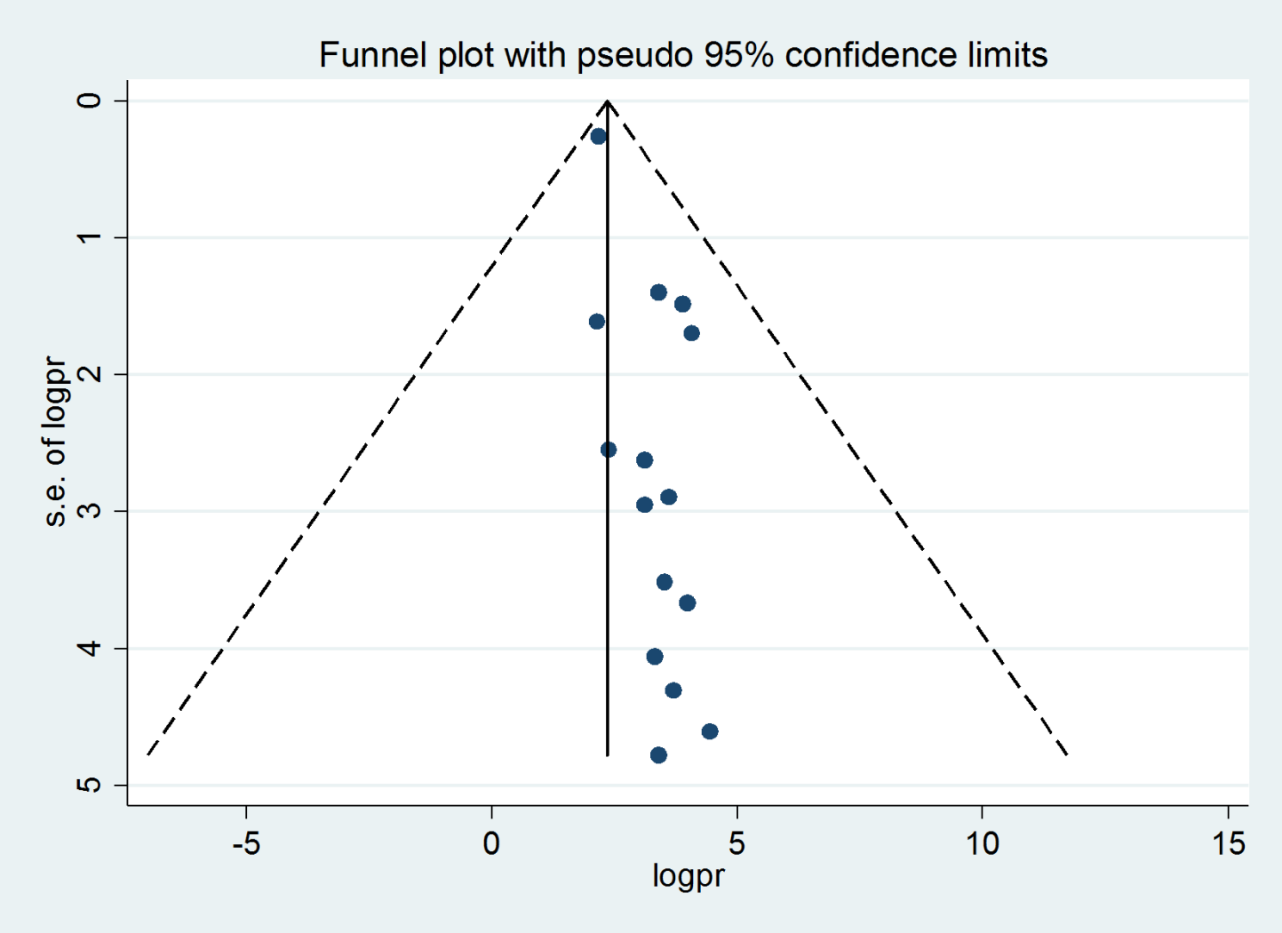
Funnel plot displaying asymmetrical distribution of studies for the presence of publication bias

**Fig. 9:**
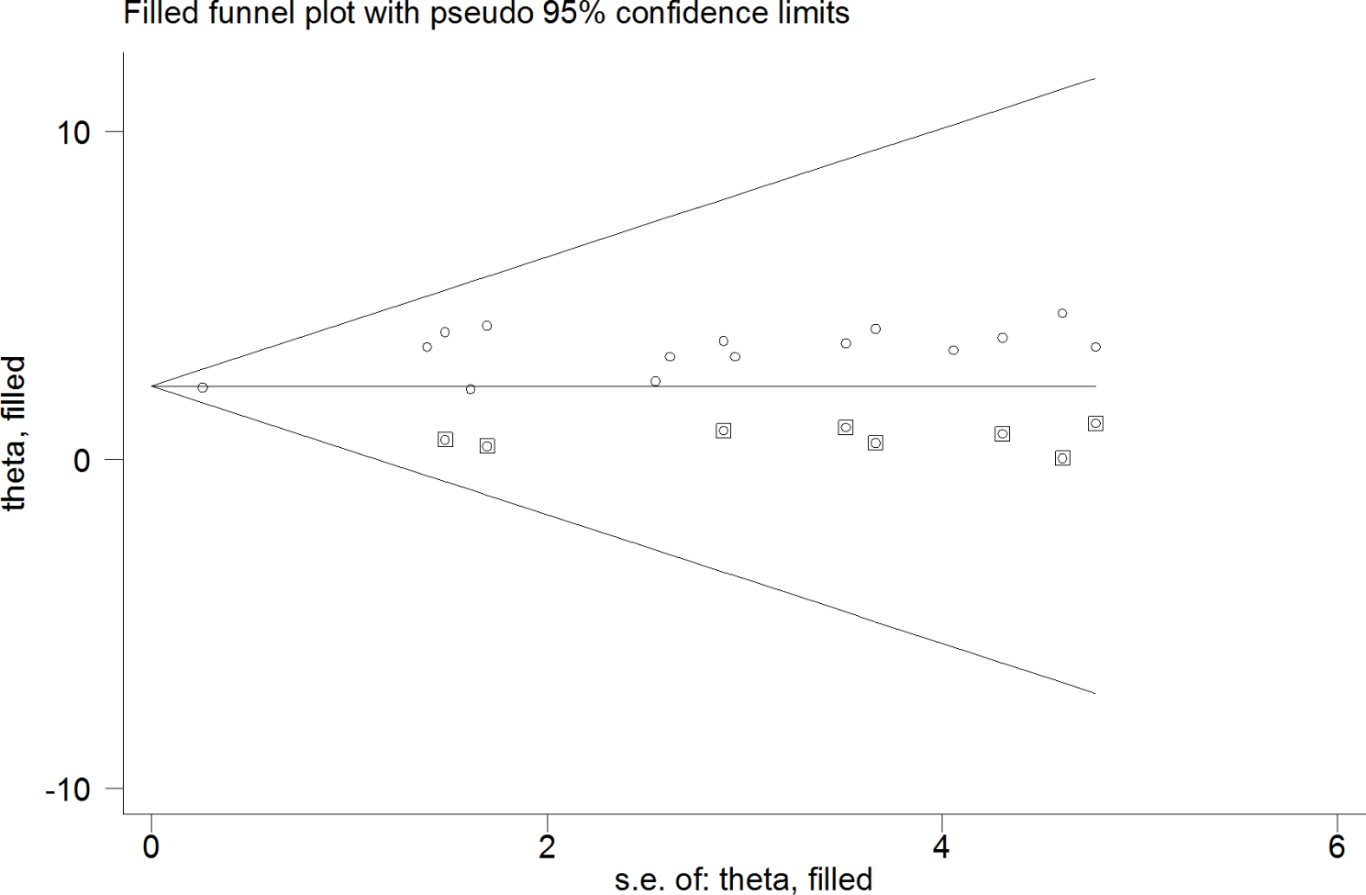
Trim and fill methods of analysis displaying the presence of eight missing studies causing for funnel plot asymmetry

**Fig. 10:**
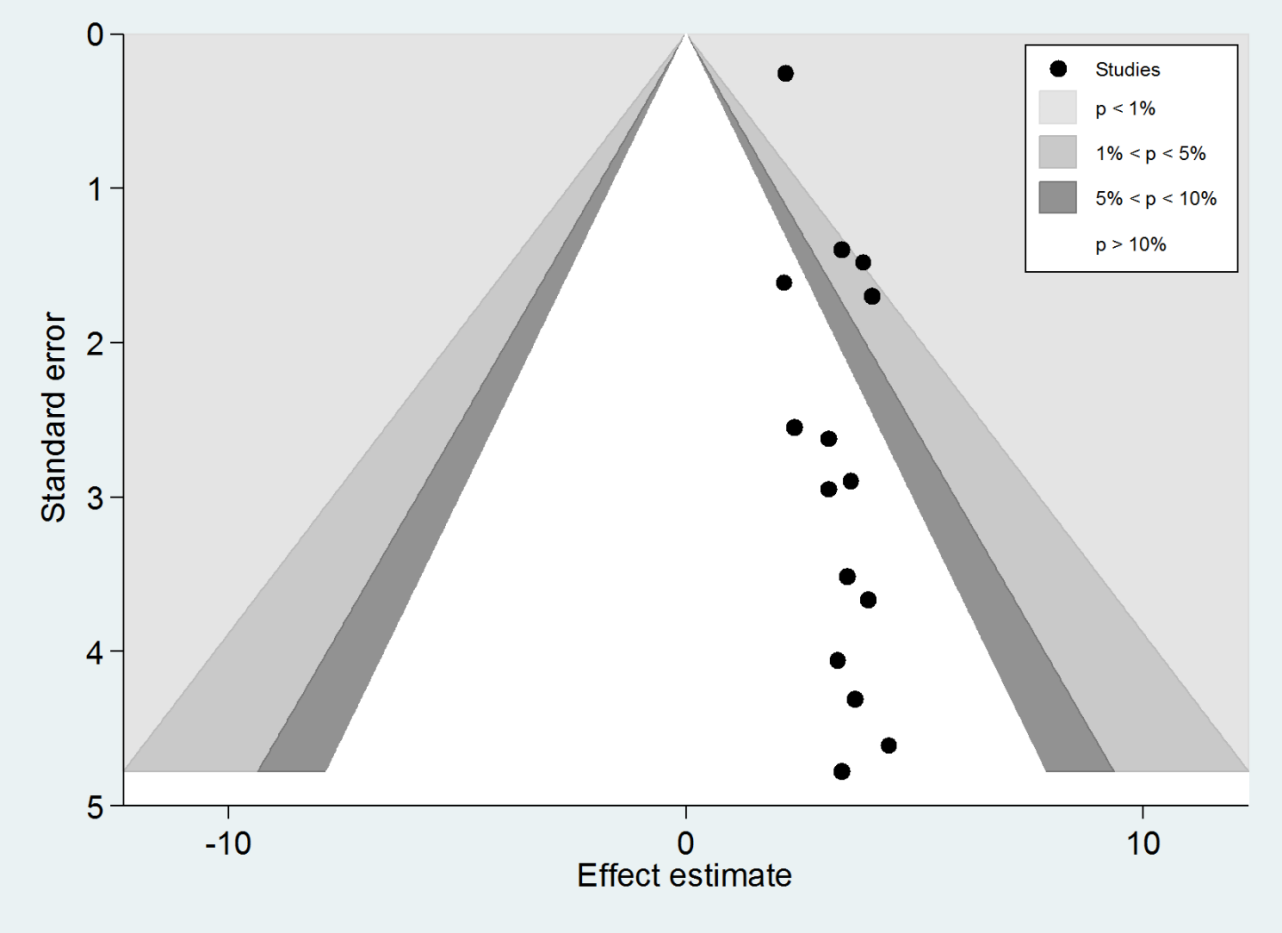
Counter-enhanced funnel plot showing that funnel plot asymmetry is due to the other factors other than the presence of publication bias

This study employed a combination of funnel plot visualization and both subjective and objective Egger’s and Begg’s tests to evaluate the presence of publication bias. The funnel plot, as depicted in Fig. 8, revealed an asymmetrical distribution of visual observation studies. Additionally, Begg’s correlation test and Egger’s regression test were conducted, yielding results of p = 0.001 and p = 0.037, respectively. These findings indicated the presence of significant publishing bias. To address this issue, a Duval and Tweedie trim-and-fill analysis was performed to correct the asymmetry observed in the funnel plot. Specifically, eight imputed studies were acquired in the trim and fill analysis to rectify the asymmetry in the funnel plot, as illustrated in Fig. 9. To further elucidate the underlying reasons for the observed asymmetry in the funnel plot, a counter-enhanced funnel plot was also conducted. The results of this analysis, as depicted in Fig. 10, suggest that publication bias is less likely to be the cause of the observed asymmetry, as the majority of the studies are located in the non-significant zone.

#### Leave –one-out-sensitivity analysis

To assess the individual impact of each study on the overall prevalence of post-abortion depression, a leave-one-out sensitivity analysis was performed. The results of this analysis indicated that the removal of any single study did not yield significant alterations in the global prevalence of post-abortion depression (Table 3).

**Table 3:** The pooled prevalence of post-abortion depression worldwide when one study omitted from the analysis a step at a time

Study omitted	Estimate	95%CI
A.A.Boersma/2014	34.82	23.21–46.44
Akdag Topal/2019	30.99	19.98-42.00
Angela J Taft/2008	34.85	22.50–47.20
Asma Sa’d Basha et.al/2020	35.37	23.59–47.15
Bujar Obertinca et.al/2016	34.98	23.33–46.63
F.Hanschmidt et.al/2017	36.22	24.32–48.12
Farnoosh Moafi/2018	33.13	21.74–44.51
Kukuluskiene/2022	32.68	22.57–42.79
L.Gao et.al/2019	34.33	22.70-45.97
L. Jacob et.al/2017	36.35	26.18–46.53
Zhang et.al/2022	35.38	23.54–47.22
Wang et.al/2021	33.67	22.60-44.33
Azin et.al/2020	34.08	22.52–45.65
Mutiso et.al/2018	34.50	22.90-46.18
Kolte et.al/2014	36.40	23.97–48.84
Combined	34.51	23.34–45.68

## Discussion

Women within the age range of childbearing are often faced with the occurrence of miscarriage and abortion, with the latter being particularly prevalent. The loss of a pregnancy can result in significant impairment for couples, particularly in the areas of physical and emotional health, general and mental well-being, vitality, and social functioning, as evidenced by previous research [50]. Such individuals may also experience “sub-syndrome depression,“ “depressive disorder,“ and “complicated grief,“ as reported in studies [51, 52]. Furthermore, maternal depression has been linked to a heightened risk of unfavorable pregnancy outcomes, including miscarriage, hypertension, preterm labor, low birth weight, and issues related to the emotional and behavioral development of the infant [53].

This study aimed to conduct a systematic review and meta-analysis to determine the prevalence of post-abortion depression on a global scale. The results indicated that the prevalence of depression among women who had undergone an abortion was 34.5% (95%CI: 23.34–45.68) worldwide. Furthermore, the study revealed significant variations in the prevalence of post-abortion depression across different regions, continents, and economic statuses. While previous systematic reviews have explored depression in women globally [54–57] or in specific geographic regions, such as Asia [58, 59] and Africa [60], this study represents the first global meta-analysis of literature on post-abortion depression, to the best of the researchers’ knowledge.

This finding revealed a higher prevalence of depression compared to previous meta-analytic research conducted in both high-income nations (13%) [54] and low- to middle-income nations (19%) [56]. However, our findings are lower than those reported in studies that have identified depression prevalence rates ranging from 1.9 to 82.1% in affluent countries and 5.2–74% in impoverished nations [61]. Furthermore, our results fall below those of a study encompassing 40 countries, which reported depression prevalence rates ranging from 0 to 60% in developing nations [62]. These observed discrepancies may be attributed to variations in sample sizes, study participants, self-report measures, and the diverse measurement types and cutoff criteria employed across the studies [63–65].

This review was limited in its scope as it did not consider all geographical regions. Specifically, the continents of North America, South America, and Antarctica were not included due to a paucity of available literature. Conversely, Asia (n = 6) and Europe (n = 7) were well-represented in the survey, while Africa (n = 1) and Australia (n = 1) were underrepresented. Consequently, the findings of this investigation revealed significant differences between continents. For example, Asia exhibited the highest prevalence of post-abortion depression (37.58%; 95% CI: 26.47–48.50), followed by Europe (32.69%; 95% CI: 14.71–50.67), Africa (34.1%; 95% CI: 27.21–32.74), and Australia (30%; 95% CI: 27.26–32.74). Additionally, the study revealed that, according to WHO regions (excluding the American and South East Asian regions), the Eastern Mediterranean region had the highest prevalence of post-abortion depression (38.94%; 95% CI: 19.34–58.54), while the European region had the lowest prevalence (32.69%; 95% CI: 14.71–50.54). This finding is consistent with a previous study conducted in Asia, which reported a range of 3.5–63.6% prevalence of depression [66].

Our findings in this meta-analysis also showed that there was a substantial variation in post-abortion depression across categories of income level, screening methods, and research design. Specifically, our results indicate that the incidence of post-abortion depression is considerably higher in lower- and middle-income countries (42.91%; 95%CI: 30.80-55.01) compared to high-income countries (24.98%; 95%CI: 10.36–39.61). This disparity may be attributed to the low social status of individuals, which can impede access to intangible resources such as security, opportunity, and education, irrespective of their objective income levels when they reside below the societal material standards [67]. The loss of certain types of social capital is believed to contribute to family dysfunction, health issues, and mood disorders.

The present study has revealed significant variations in the prevalence of post-abortion depression across different diagnostic methods. The Center for Epidemiological Studies Depression Scale has exhibited the highest prevalence rate of post-abortion depression (30%; 95% CI: 27.37–32.63), while the lowest prevalence rate (18.53%; 95% CI: 10.66–26.40) was observed with any other screening measure. Furthermore, cross-sectional studies have reported a higher prevalence rate of depression (36.42%; 22.61–50.23) compared to cohort studies, which have indicated a lower frequency of depression (22.72%; 95%CI: 5.63–39.88). It is noteworthy that cross-sectional studies are more efficient in determining prevalence rates. However, further evidence is required to support this statistical variation.

The results mentioned above possess significant implications, as the manifestation of depressive symptoms among healthcare providers underscores the pressing need for expeditious and effective aid to mitigate the persistent effects of these stressors. It may be crucial to provide healthcare providers with tailored coping mechanisms, in addition to furnishing them with supplementary resources such as counseling and opportunities for respite from their professional obligations. As evidenced by extant literature, the absence of such support may engender unfavorable outcomes, including compromised quality of care and burnout [68], which may have long-lasting ramifications and contribute to a depleted workforce.

This meta-analysis also holds significant importance in bolstering global public health efforts and bridging the knowledge gap in the treatment of mental health disorders [69]. The findings of this study can serve as a valuable resource for stakeholders and governments to facilitate sustainable development in mental health by promoting the prioritization and allocation of resources toward mental health initiatives.

In this study, a random-effects model was employed to account for the significant variance in between-study heterogeneity. A leave-one-out sensitivity analysis was conducted, which indicated that no individual study had a significant impact on the overall prevalence of post-abortion depression. To further investigate the presence of heterogeneity, sub-group analyses were performed based on the continent, WHO area, depression screening method, income level, and study design. The observed heterogeneity may be attributed to differences in sample demographics, paper characteristics, or socio-cultural factors.

## Conclusion

In summary, the occurrence of post-abortion depression was found to be highly prevalent. Furthermore, the prevalence of post-abortion depression exhibited variation based on geographical location, screening methodology, income level, and research design. Consequently, post-abortion depression was observed to be more prevalent in the WHO Eastern Mediterranean region and Asia. The utilization of the Center of Epidemiological Studies Depression Scale and cross-sectional study design revealed that depression was more prevalent in countries with lower-middle income. Individuals who have undergone an abortion should receive additional care and psychological support from healthcare providers, as well as their spouse, family, and community.

### Strengths and limitations of the review

The study does have some merit. The present study holds significant value as it utilized global compressive electronic search engines to initiate the investigation. The study also aimed to determine the prevalence of post-abortion depression on an international and regional scale. Furthermore, the study demonstrated the variation of depression across income status and the diagnostic tool. However, the study is not without limitations. The absence of a meta-analysis study with the same population makes it challenging to compare the results. It is crucial to note that there is a possibility of misclassification due to the inconsistent diagnostic cut-off criteria applied to the measurement tools in this evaluation. Additionally, the lack of articles published in languages other than English and the absence of data from certain geographic areas that correspond to WHO regions and World Bank income groups, such as the American and South East Asian regions, as well as low-income countries, are also limitations of the stud.

The I^2^ statistic is commonly used to indicate a significant level of heterogeneity. However, it is crucial to recognize that the I-square statistic may not always serve as an unambiguous indicator of heterogeneity, as the source of heterogeneity may be attributed to the specific command utilized (in this case, the “Metan” command).

The study has identified the existence of publication bias, as objectively ascertained through the implementation of Egger’s regression test. It is crucial to exercise prudence in the interpretation of the findings of this investigation for informed decision-making and resource allocation, as the evidence may have been impacted by bias.

Self-reported responses are susceptible to exaggeration, as respondents may feel too ashamed to disclose personal information, and various biases may influence the outcomes, including social desirability bias. Social desirability bias is a phenomenon where individuals are either consciously or unconsciously influenced to report experiences that are deemed socially acceptable or desirable. Consequently, the outcomes may be either underestimated or overestimated.

### Electronic supplementary material

Below is the link to the electronic supplementary material.

Additional file 1: S1_File.Prisma checklist.

Additional file 2: S2_File. Methodological quality assessment of included studies using Joanna Brigg’s Institute quality appraisal criteria scale (JBI). The eight-item questions assessing inclusion criteria, study setting and participant, exposure measurement, objectives, confounder, statically analysis, outcome measurement, and dealing confounder were used.

Additional file 3: S3_File. Risk of bias assessment for the included studies. The ten-item questions of which four items assess external and six items assess internal validity were used.

## Data Availability

All relevant data are within the Manuscript and its Supporting Information files.

## Abbreviations

CES-D: Center of Epidemiological Studies Depression Scale
EPDS: Edinburgh Postnatal Depression Scale
HADS: Hospital Anxiety Depression Scale
PHQ-9: Patient Health Questionnaire 9
SDS: Self-rating Depression Scale
