# Supplementary material for: Global prevalence of post-abortion depression: systematic review and Meta-analysis
Source: BMC Psychiatry. 2023 Oct 26;23:786. doi: 10.1186/s12888-023-05278-7 (PMC10605843; doi:10.1186/s12888-023-05278-7)
Supplement: Supplementary file 1 — Additional file 1: S1_File.Prisma checklist. [file 12888_2023_5278_MOESM1_ESM.docx]

File 2. Quality assessment for the included Studies

| Item | Clearly defined inclusion | Describe study setting and participant | Valid and reliable exposure measurement | | Objective and standard criteria for measurement | Identified confounder | Strategies to deal with confounders | | Valid and reliable outcome measurement | Appropriate statically analysis | | No of ‘yes’s ‘ | |
| --- | --- | --- | --- | --- | --- | --- | --- | --- | --- | --- | --- | --- | --- |
| Akdag Topal | Yes | Yes | No | | Yes | Yes | No | | Yes | Yes | | 6/8=75 | |
| Asma Sa’d Basha et.al | Yes | Yes | Yes | | Yes | No | No | | Yes | Yes | | 6/8=75 | |
| F.Hanschmidt et.al | Yes | Yes | No | | Yes | Yes | Yes | | Yes | Yes | | 7/8=87.5 | |
| Bujar Obertina et.al | Yes | Yes | Yes | | Yes | Yes | No | | Yes | Yes | | 7/8=87.5 | |
| Farnoosh Moafi | Yes | Yes | No | | Yes | Yes | Yes | | Yes | Yes | | 7/8=87.5 | |
| Kolte et.al | Yes | Yes | No | | Yes | Yes | No | | Yes | Yes | | 6/8=75 | |
| Kukulskiene | Yes | Yes | Yes | | Yes | Yes | No | | Yes | Yes | | 7/8=87.5 | |
| L.Gao et.al | Yes | Yes | Yes | | Yes | No | No | | Yes | Yes | | 6/8=75 | |
| Zhang et.al | Yes | Yes | No | | Yes | Yes | Yes | | Yes | Yes | | 7/8=87.5 | |
| Azin et.al | Yes | Yes | Yes | | Yes | Yes | No | | Yes | Yes | | 7/8=87.5 | |
| Mutiso et.al | Yes | Yes | No | | Yes | Yes | Yes | | Yes | Yes | | 7/8=87.5 | |
| **For case–control** | | | | | | | | | | | | | |
| Criteria | | | | Wanga et.al | | | | | | | | |  |
| Compilations of cases and control | | | | Yes | | | | | | | | |  |
| Matched case and controls | | | | Yes | | | | | | | | |  |
| The same criteria used to identify case and control | | | | Yes | | | | | | | | |  |
| Standard, valid and reliable measurement of exposure | | | | Yes | | | | | | | | |  |
| The same measurement of exposure for case and control | | | | Yes | | | | | | | | |  |
| Identifying confounder | | | | No | | | | | | | | |  |
| Strategies to identify cofounders | | | | Yes | | | | | | | | |  |
| Standard, valid and reliable ways to assess outcomes for case and control | | | | Yes | | | | | | | | |  |
| Long enough period of exposure | | | | No | | | | | | | | |  |
| Appropriate statistical analysis | | | | Yes | | | | | | | | |  |
| Percentage of yes (%) | | | | 8/10=80% | | | | | | | | |  |
| **For prospective cohort** | | | | | | | | | | | | | |
| Criteria | | | | | | L.Jacobo et.al | | Angela J Taft | | | A.A Boersma | | |
| Two groups are similar and recruited from the same population | | | | | | Yes | | Yes | | | Yes | | |
| Similar measurement of exposure both for exposed and  unexposed groups | | | | | | Yes | | Yes | | | Yes | | |
| Valid and reliable measurement of exposure | | | | | | Yes | | Yes | | | Yes | | |
| Identifying confounders | | | | | | Yes | | Yes | | | Yes | | |
| Strategies to deal with confounders | | | | | | No | | No | | | No | | |
| Groups are free of the outcomes at the beginning | | | | | | Yes | | Yes | | | Yes | | |
| Valid and reliable measurement of outcomes | | | | | | Yes | | Yes | | | Yes | | |
| Long enough follow-up time for the occurrence of outcomes | | | | | | Yes | | Yes | | | No | | |
| Complete follow-up time | | | | | | Yes | | Yes | | | Yes | | |
| Strategies to address lost follow-up | | | | | | Yes | | No | | | No | | |
| Percentage of yes (%) | | | | | | 9/10=90% | | 8/10=80% | | | 7/10=70% | | |
