# Supplementary material for: Global prevalence of post-abortion depression: systematic review and Meta-analysis
Source: BMC Psychiatry. 2023 Oct 26;23:786. doi: 10.1186/s12888-023-05278-7 (PMC10605843; doi:10.1186/s12888-023-05278-7)
Supplement: Supplementary file 3 — Additional file 3: S3_File. Risk of bias assessment for the included studies. The ten-item questions of which four items assess external and six items assess internal validity were used. [file 12888_2023_5278_MOESM3_ESM.docx]

File3. Assessment of risk of bias for the included studies

| Item | External validity | | | | Internal validity | | | | | |  | |
| --- | --- | --- | --- | --- | --- | --- | --- | --- | --- | --- | --- | --- |
|  | Representativeness s of the target population | Representativeness s of the sampling frame | Radom sampling g or census | Minimal response e bias | Data were collected d directly | Acceptable e case definition used in the study | Valid and reliable measurement t | The same mode of data collection n for all study subject | Appropriate e length of prevalence period for parameter of interest | Appropriate numerators and denominator s of interest | No of yes | Summary of risk of bias |
| Akdag Topal | Yes | Yes | No | Yes | Yes | No | Yes | Yes | Yes | Yes | 8 | Low-  risk |
| Asma Sa’d Basha et.al | Yes | Yes | Yes | Yes | No | No | Yes | Yes | Yes | Yes | 8 | Low risk |
| F.Hanschmidt et.al | Yes | Yes | No | Yes | Yes | No | Yes | Yes | Yes | Yes | 8 | Low- risk |
| BujarObertina et.al | Yes | Yes | No | Yes | Yes | Yes | Yes | Yes | Yes | Yes | 9 | Low risk |
| Farnoosh Moafi | Yes | Yes | No | Yes | Yes | Yes | Yes | Yes | Yes | Yes | 9 | Low- risk |
| Kolte et.al | Yes | Yes | Yes | Yes | Yes | No | Yes | Yes | Yes | Yes | 9 | Low- risk |
| Kukulskiene | Yes | Yes | No | Yes | Yes | Yes | Yes | Yes | Yes | Yes | 9 | Low- risk |
| L.Gao et.al | Yes | Yes | Yes | Yes | No | No | Yes | Yes | Yes | Yes | 8 | Low- risk |
| Zhang et.al | Yes | Yes | No | Yes | Yes | Yes | Yes | No | Yes | Yes | 8 | Low- risk |
| Azin et.al | Yes | Yes | Yes | Yes | Yes | No | Yes | Yes | Yes | Yes | 9 | Low- risk |
| Mutiso et.al | Yes | Yes | Yes | Yes | No | No | Yes | Yes | Yes | Yes | 8 | Low- risk |
| Wanga et.al | Yes | Yes | No | Yes | Yes | Yes | Yes | Yes | Yes | Yes | 9 | Low – risk |
| L.Jacobo et.al | Yes | Yes | No | Yes | Yes | No | Yes | Yes | Yes | Yes | 8 | Low-risk |
| Angela J Taft | Yes | Yes | No | Yes | Yes | No | Yes | Yes | Yes | Yes | 8 | Low-risk |
| A.A Boersma | Yes | Yes | No | Yes | Yes | Yes | Yes | Yes | Yes | Yes | 9 | Low risk |
